# Limb conservation surgery in biphasic synovial sarcoma of thigh with vascular involvement: A race against time

**DOI:** 10.1016/j.ijscr.2023.108646

**Published:** 2023-08-09

**Authors:** Sagun Ghimire, Pashupati Pokhrel, Samir Thapa

**Affiliations:** a KIST Medical College and Teaching Hospital, Lalitpur, Nepal; b Maharajgunj Medical Campus, Kathmandu, Nepal

**Keywords:** Synovial sarcoma, Limb conservation surgery, Vessel graft

## Abstract

**Introduction:**

Synovial sarcomas are malignant soft tissue neoplasm representing 5 to 10 % of all Soft tissue sarcoma with incidence of 2.75 per 100,000. It is presented in particular along with extra articular location with no as such relation to synovial structures. Among various histological pattern biphasic synovial sarcoma (SS) is considered classical type. Involvement of neurovascular structures in synovial sarcoma is least noted and such invasion by malignant cells is considered an indication for amputation. However vessel reconstructive surgeries have also resulted in conservation of limb hence providing good quality of life.

**Case presentation:**

13 years old Asian female presented with complaint of exposed synthetic vessel graft from her previous surgical site where femoral vessel reconstruction was carried out for SS of thigh with femoral vessel involvement. Following her (wide local excision) WLE and femoral vessel reconstruction in another center she suffered thrombosis of her femoral vessel following which emergency thrombectomy was also carried out. Later in our center vertical rectus abdominis myocutaneous flap (VRAM) flap was used to manage her exposed synthetic graft. At subsequent follow up patient was ambulating well with no signs of radiological metastasis.

**Clinical discussion:**

Surgical resections of mass along with synthetic graft placement of the concomitant vascular bundle have also shown significant improvement in reducing the burden of the severe diseases such as synovial sarcoma.

**Conclusion:**

SS with its malignant pathophysiology have impacted severely the quality of life of even among the pediatrics group of population. It is utmost need to set up proper and definitive muscular oncological care to reduce the morbidity and mortality associated with such malignancies.

## Highlights

• Synovial sarcomas are malignant soft tissue neoplasm representing 5 to 10 % of all Soft tissue sarcoma
• Involvement of neurovascular structures in SS is least noted and such invasion by malignant cells is considered an indication for amputation.
• Surgical resections of mass along with synthetic graft placement of the concomitant vascular bundle have also shown significant improvement in reducing the burden of the severe diseases such as synovial sarcoma.
• Immunohistochemically with specific tumor markers like TLE, CD99 is the main stay of diagnosis further aided by histopathology and radiological examination
• advancements in radiological diagnostic modalities along with implication of chemotherapy and vascular reconstruction have become a forefront in conservation of the affected extremity

## 1 Introduction

Synovial sarcomas are malignant, high-grade, soft-tissue neoplasms with estimated representation of 5 % to 10 % of all soft tissues sarcomas along with incidence of 2.75 per 100,000 among the general population [1,2]. It is located in the vicinity of the joints, tendons and bursa but contrary to what its name might suggest it is also prevalent in extra articular locations with no apparent relation to synovial structures as well [3]. Histologically biphasic pattern synovial sarcoma is considered the classic type well appreciated by the coexistence of morphologically different but genetically similar epithelial cells and fibroblast-like spindle cells separating it from monophasic and poorly differentiated patterns of synovial sarcoma [1]. Immunohistochemistry with specific tumor markers like TLE, CD99 is the main stay of diagnosis further aided by histopathology and radiological examination [4]. In 5 % to 10 % soft tissue sarcoma of extremity involvement of femoral vessels are most frequent ones regarding vascular involvement [5]. Surgery is considered the mainstay treatment modality where excision of the tumor mass with the margins of the healthy tissue is carried out [6]. The extension of tumor up to great vessels and nerves in lower extremity was previously designated as indication for limb amputation but recent studies have discovered equivalent oncological outcome and superior functional outcome in limb sparing surgery with vessel reconstruction [7]. This case report has been written according to SCARE guideline [8].

## 2 Case summary

### 2.1 Patient information

**Fig. 1:**
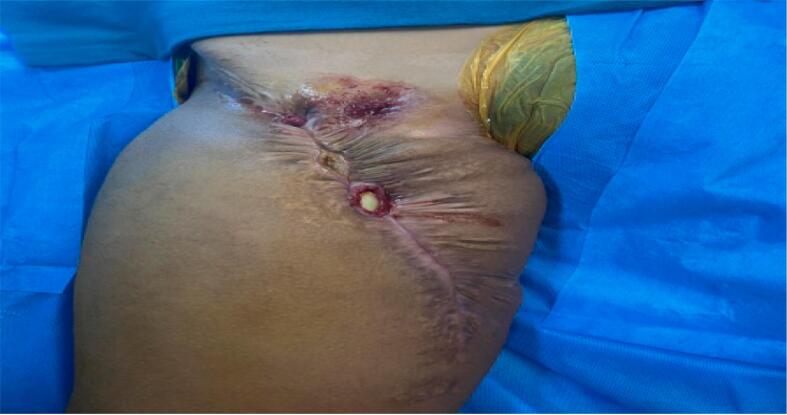
Exposed synthetic femoral vessel graft from previous surgical site of Wide local excision and femoral vessel reconstruction.

We are demonstrating the case of a 13-year-old previously healthy Asian female, who was admitted through our hospital's department of orthopedics with a chief complaint of exposed synthetic femoral vessel graft (Fig. 1). It was in the form a visible white synthetic graft exposed through her scar from her previous surgical procedure in the anteromedial aspect of her right thigh. It wasn't associated with local signs of inflammation, such as redness, hotness, swelling or discharge. Moreover, it wasn't associated with neither limb paresthesia nor coldness of the diseased extremity but there was decreased oxygen saturation when measured in her right foot. There was no report of intermittent claudication, night or cold sweats, fever, weight changes, malaise, general weakness, or overlying skin changes. Furthermore, there were no changes in bowel habits or noteworthy genitourinary symptoms. Her Body Mass Index was 22 kg/m^2^. Her surgical, family, drug, psychosocial, and allergic histories were negative. Before visiting to our center with the current complaint she presented to other two different centers, first with the chief complaint of swelling and feeling of lumpiness over right thigh for three years. She developed swelling over anteromedial aspect of right thigh which was progressively increasing and associated with pain. Pain was on and off, non-radiating, dull aching and aggravated during walking without any relieving factors. Second center for wide local excision and femoral vessel reconstruction for synovial sarcoma of her right thigh with vascular involvement and in the third center emergency thrombectomy for synthetic femoral graft thrombosis and following which after 1 year she presented to our center with current complaint.

### 2.2 Clinical findings

During her current visit to our center vital signs measurements yielded normal readings except oxygen saturation in her right leg showed fluctuating reading between 80 % and 85 %. Via inspection, no noted skin ulceration, overlying skin discoloration, ecchymosis, pin-point spotting, cyanosis, or pallor. Via palpation, a relatively deep solid mass was palpated in the anterolateral aspect of her right thigh near to lateral aspect of her right patella. It was painless, immobile, and exerted no overlying skin movement, and no palpable bruits were demonstrated. The arterial pulses were palpable along the entirety of the arterial axis of both lower limbs. Through auscultation, no thrills were heard over the mass or along the ipsilateral arterial access. During her first visit where she underwent excisional biopsy, on clinical examination there was around 10 cm × 5 cm painless, immobile mass, without any overlying skin movement and skin changes. There was associated pain which was on and off, non-radiating, dull aching and aggravated during walking without any relieving factors. On the second center where she suffered thrombosis of synthetic femoral vessel graft as a complication after WLE with femoral vessel reconstruction, clinical findings showed On the 5th post-operative day following her surgery she complaint of pain on the surgical site and local examination of right leg showed generalized swelling over right thigh and leg, pale overlying skin, low temperature of right leg compared to left, absent active toe movement, sensation not intact, capillary refill time (CRT) not appreciated and arterial dorsalis pedis, anterior tibial artery, posterior tibial artery not palpable.

### 2.3 Laboratory investigations

The entirety of preoperative laboratory panel revealed normal results.

### 2.4 Diagnostic assessment

During her presentation to the first center with swelling and pain in the right thigh, she underwent MRI of the pelvis + right thigh which showed relatively well defined heterogeneous T1 iso to low T2 intermediate to thigh and STIR high signal intensity mass in intermuscular plane of anteromedial aspect of right thigh encasing superficial femoral vessels. Immunohistochemistry studies i.e. H and E, KI-67 (MIB-1), CD99 (EP8), TLE (1F5) showed biphasic tumor composed of spindle cells and gland containing mucin. The cells show moderate cytoplasm, round to oval nuclei and prominent nuclei, bony spicules entrapped amidst it. The neoplastic cells express TLE, CD99, Ki-67 proliferative index was 20 %. Histopathology report after her Wide local excision (WLE) with femoral vessels reconstruction with synthetic graft and biopsy reported noted spindle cells arranged in fascicles, moderate amount of cytoplasm, elongated nuclei, and vesicular nuclear chromatin with small nucleoli in some of the cells along with tumor cells infiltrating the skeletal muscles. Adjacent areas show necrosis surrounded by foreign body giant cells. Perivascular invasion was noted but no lymphovascular invasion noted. Plain and contrast CT scan of chest and abdomen showed no any signs of metastasis. On the fifth post-operative day after her WLE and femoral vessel reconstruction there was suspected clinical findings of synthetic graft thrombosis hence peripheral venous Doppler study was done which showed cord like echogenicity in the wall of external iliac vein proximal to the venous graft with no flow in color and spectral Doppler study suspicious of thrombosis hence CT angiogram of lower limbs was done which showed thrombosis of right common femoral vein and right superficial femoral vein with thin peripheral rim of opacification. Preliminary patient preparation for her closure of exposed synthetic vessel graft was comprised of maintaining a nil-per-mouth patient status, setting-up intravenous access, administrating suitable preoperative intravenous antibiotics, blood sampling and cross match to prepare for surgical intervention. Notable obstacles were the device unavailability of a Positron Emission Tomography (PET) scan.

### 2.5 Therapeutic intervention

**Fig. 2:**
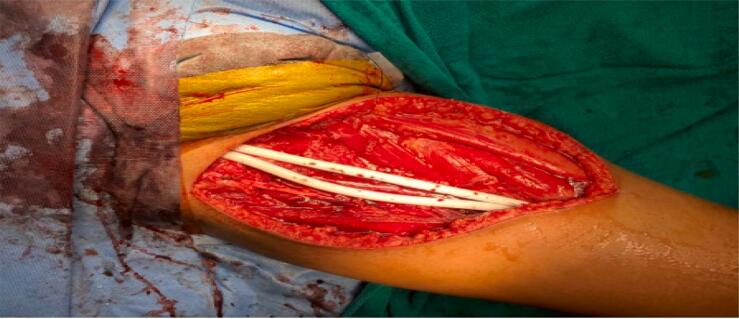
Synthetic expanded ploytetrafluoroethylene (ePTFE) Protease graft connecting superficial femoral artery and vein with supra geniculate popliteal artery and vein.

**Fig. 3:**
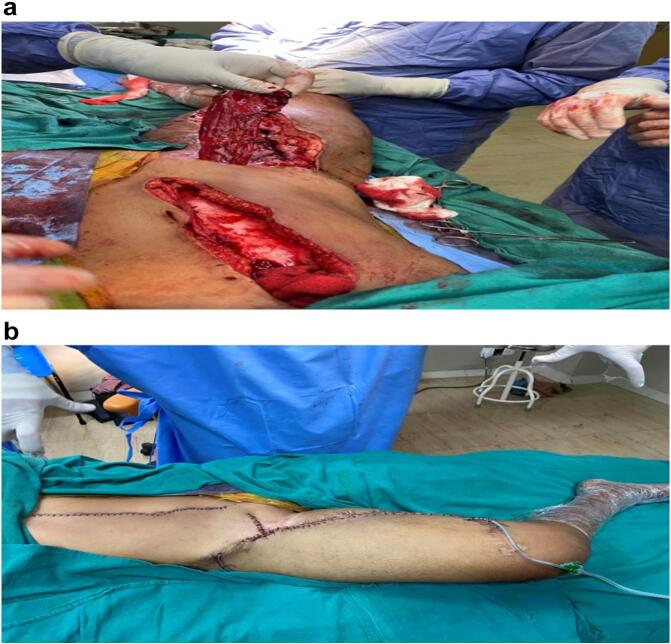
a Removal of vertical rectus abdominis myocutaneous (VRAM) flap. b Surgical site after placement of vertical rectus abdominis myocutaneous (VRAM) flap.

**Fig. 4:**
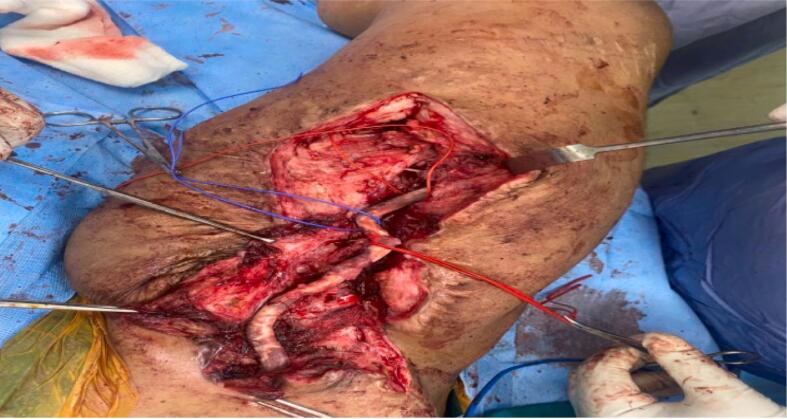
Removal of synthetic vein graft and thrombectomy of synthetic artery graft using fogarty catheter.

Taking all the known factors into consideration, surgical intervention was warranted. During her surgical treatment in the first center she presented, she underwent excision and biopsy. After confirmation of her diagnosis of synovial sarcoma of right thigh she underwent wide local excision (WLE) and femoral vessel reconstruction. The surgery took place at a tertiary hospital with coordination among department of orthopedics and department of cardiothoracic and vascular surgery. Furthermore, it was carried-out under general anesthesia with no perioperative complications whatsoever. A longitudinal incision along the medial side of the right thigh was performed. The mass was then isolated from its surrounding structures. Concomitant Superficial Femoral Artery and Vein infiltration by the neoplasm was noted, thus, the affected segments were resected along with the mass with adequate free margins. Afterwards, a bypass graft of said artery and vein with a synthetic Expanded Polytetrafluoroethylene (ePTFE) protease graft connecting the Superficial Femoral Artery and Vein with the Supra-geniculate Popliteal Artery and Vein, respectively was performed (Fig. 2). On the 5th post-operative day emergency thrombectomy was performed for thrombosis of synthetic vessel graft where using fogarty catheter thrombus was removed from proximal and distal end of graft. Capillary refill time (CRT) and temperature over distal limb examined which was normal and hence Graft was closed with prolene. During her current visit to our center closure of exposed graft using vertical rectus abdominis myocutaneous flap (Fig. 3a-b) and synthetic vein graft was removed and thrombectomy of synthetic artery graft using fogarty catheter was done (Fig. 4). Post operatively she was kept under observation in intensive care unit and no any dreadful complications were present. Excisional biopsy taken from right thigh during this operation showed no dysplasia/malignancy. On her 10th post-operative day real time B mode grey scale ultrasound and color Doppler of the arterial system of the right lower leg was done which showed reduced flow below the distal part of superficial femoral artery and reduced flow in posterior tibial, peroneal and anterior tibial artery suggestive of thrombus or obstruction in proximal segment hence warfarin was started. Said patient had a satisfactory postoperative surgical recovery and as a result, she was discharged within 2 weeks of her operation. We provided her with protocol directives, which aided her recovery (i.e., daily sterile dressings of her wound, suitable analgesia for any concomitant pain, and an appropriate prescription of antibiotics). Follow-up surveillance is still being carried out in the outpatient clinic for 2 months so far. We scheduled her regular visitations at our tertiary care center hospital clinic to thoroughly conduct physical examination and observe her functional outcome along with radiological assessment for exclusion of recurrence and metastasis. All of which yielded normal results.

## 3 Discussion

Sabrazes et al. in 1934 coined synovial sarcomas as neoplasms that originates from mesenchymal cells with tendency to originate from juxta articular areas of lower extremity [9]. The incidence rate of synovial sarcoma in the pediatric population is 0.81 per 1 million whereas for adult population it is 1.42 per 1 million [3]. It is frequently considered misnomer that synovial sarcoma is the sarcoma of the synovium, where as it is not a sarcoma of synovial origin. It merely depicts histopathological resemblance with the synovial tissue. The origin of synovial sarcoma from pluripotent stem cells which possess the capability to differentiate into mesenchymal and/or epithelial tissues and cannot differentiate into synovial tissue still holds strong [1,10]. Primarily synovial sarcoma presents in the forms of soft tissue mass but clinically doesn't manifest in fashion similarly to soft tissue sarcomas where a rapidly proliferating sizeable mass is appreciated [11,12]. Rather, maximum number of SSs present as slowly growing masses with 2 years of average growth period prior to the patient's first established diagnosis [13]. Clinically when compared with other forms of soft tissue tumors SSs has considerably longer duration of symptoms ranging from pain, swelling and feeling of lumpiness around the extremities [14]. Morphologically Synovial sarcoma has a variety of patterns, but its chief forms are the classic biphasic pattern, of glandular or solid epithelial structures with monomorphic spindle cells and the monophasic pattern, of fascicles of spindle cells with only immunohistochemical or ultrastructural evidence of epithelial differentiation [15].

Radiological method of diagnosis acts as additional aid in increasing the diagnostic validity and MRI with or without contrast is considered superior to CT scan for well appreciation of lesion [16]. Moreover in defining accurate diagnosis of synovial sarcomas (SSs) the role of immunohistochemistry still holds as robust diagnostic modality further aiding to exclude other differential diagnosis and diagnostic decision from the stand point of histopathological findings only is considered insufficient [12]. High affinity of synovial sarcoma for stains such as keratin, EMA, BCL-2, Vimentin, CD99 and TLE1 acts as cornerstone in accurate diagnosis of SSs [17]. In our case also the neoplastic cells showed affinity towards TLE1 and CD99.

Exceptionally malignant tendency of SSs to metastasize primarily to lungs, lymph node and bone marrow followed by distant metastasis in 13 % of patients [18]. The most acceptable treatment modality for SSs is surgical resection of tumor along with wide negative margins supplemented by adjuvant therapy based upon patient's individual growth and characteristics [19]. Neoadjuvant chemotherapy is increasingly used to limit loss of function after wide surgical excision with the ultimate aim of improving patient survival. Neoadjuvant epirubicin and ifosfamide improves survival of patients affected by five high-risk soft tissue sarcoma histologies of trunk and extremities, including undifferentiated pleomorphic sarcoma, myxoid liposarcoma, synovial sarcoma, malignant peripheral nerve sheath tumors, and leiomyosarcoma. Adjuvant systemic therapies have been tested to reduce the risk of metastatic spread after surgery with or without radiotherapy in several randomized controlled trials (RCTs). Anthracycline-based regimens using doxorubicin as the main chemotherapeutic agent were used in early studies, while more recent trials have tested anthracycline combined with ifosfamide. These treatment strategies offer a survival benefit to patients ranging between 5 % and 10 %, which has been considered unsatisfactory particularly when balanced against high-grade toxicity [19,20]. Throughout history when soft tissue sarcoma (STS) or synovial sarcoma is diagnosed in an extremity, amputation of the said limb was considered ideal management. Along with it further involvement of neurovascular structures by the tumor was considered strict indication for limb amputation [20]. However with advancements in radiological diagnostic modalities along with implication of chemotherapy and vascular reconstruction have become a forefront in conservation of the affected extremity through limb-salvage surgical interventions [20]. The foremost objective of limb-salvage surgical techniques in managing synovial sarcoma is to reach optimal control over the neoplastic situation of the patient whilst maintaining an extremity with adequate functioning and best functional outcome hence improving the quality of life of the patients. About 15 % to 25 % patients of synovial sarcoma with vascular reconstruction undergoes amputation surgeries whereas only 5 % patients undergoes limb salvage surgeries. Along with it various major dreadful complications like deep vein thrombus (DVT), pulmonary embolism and flap devascularization have resulted in further decreased the incidence of limb salvage surgeries [21]. But in our patient there was successful limb salvage with good functional outcome despite post-operative complications.

In western literature limb salvage surgery in cases of synovial sarcomas with vascular involvement is a well-established procedure. Post-operative morbidity, fear of oncological outcome and complexity of the procedure associated with vascular reconstruction has deterred many to undertake limb salvage in this subset of patients in countries of south Asia despite being reported in western literature. In our experience soft tissue sarcomas with vascular involvement are inherently very aggressive tumors with variable presentation. Resection usually involves large amount of tissue resulting in much higher incidence of wound related and post-operative complication. Reconstruction of the resected vessels possesses its own immediate postoperative issues and vascular insufficiency in long term. Because of these factors functional outcome can be unpredictable but in our case limb salvage along with vascular reconstruction despite post-operative vessel thrombosis complication have resulted in good functional outcome and noteworthy quality of life of patient.

## Consent

Written informed consent was obtained from the patient's parents(mother) for publication and any accompanying images. A copy of the written consent is available for review by the Editor-in-Chief of this journal on request.

## Ethical approval

It is exempted at my institution KIST Medical college and teaching hospital. We don't need to take approval from ethical committee for case report.

## Funding

N/A.

## Author contribution

Conceptualization: Sagun Ghimire.

Clinical diagnosis and patient management: Pashupati Pokhrel.

Manuscript review: Samir Thapa.

Writing original draft: Sanjit Kumar Shah and Avish Shah.

All authors were involved in reviewing, editing, supervision and in preparing the final manuscript.

## Guarantor

Sagun Ghimire.

## Registration of research studies

Not applicable.

## Declaration of competing interest

N/A.
